# Inverted pendular running: a novel gait predicted by computer optimization is found between walk and run in birds

**DOI:** 10.1098/rsbl.2010.0256

**Published:** 2010-05-19

**Authors:** James Richard Usherwood

**Affiliations:** Structure and Motion Laboratory, The Royal Veterinary College, North Mymms, Hatfield, Herts AL9 7TA, UK

**Keywords:** walk, run, transition, GIPR

## Abstract

Idealized models of walking and running demonstrate that, energetically, walking should be favoured up to, and even somewhat over, those speeds and step lengths that can be achieved while keeping the stance leg under compression. Around these speeds, and especially with relatively long step lengths, computer optimization predicts a third, ‘hybrid’, gait: (inverted) pendular running (Srinivasan & Ruina 2006 *Nature* 439, 72–75 (doi:10.1038/nature04113)). This gait involves both walking-like vaulting mechanics and running-like ballistic paths. Trajectories of horizontal versus vertical centre of mass velocities—‘hodographs’—over the step cycle are distinctive for each gait: anticlockwise for walk; clockwise for run; figure-of-eight for the hybrid gait. Both pheasants and guineafowl demonstrate each gait at close to the predicted speed/step length combinations, although fully aerial ballistic phases are never achieved during the hybrid or ‘Grounded Inverted Pendular Running’ gait.

## 1. Introduction

**Figure 1.**
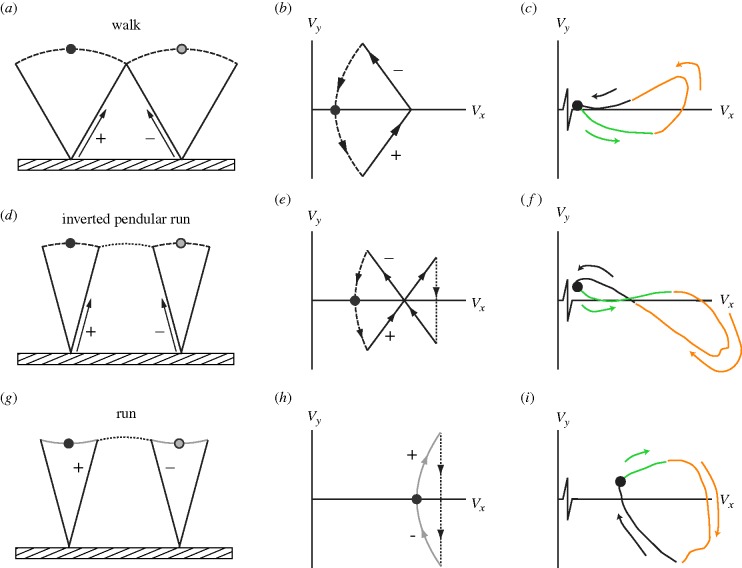
Idealized cartoons and hodographs, and example empirical (pheasant) hodographs of (*a*–*c*) walking, (*d–f*) inverted pendular running and (*g–i*) running. Black arrows relate to momentary impulses; dashed lines to passive vaulting; dotted lines to ballistic falling. Note that, while the cartoons and hodograph sketches for walking and inverted pendular running approach those for ideal, impulsive gaits, the running figure includes a qualitative realism of finite limb forces and stance paths (grey lines): running is not impulsive, stance periods are finite and the hodograph is not vertical during the leg impulse. In each case, the step cycle begins at the black circle, and the cartoons progress from left to right. +/− symbols indicate gain and loss of energy, respectively. Colour in the empirical hodographs show progression through time: green, orange, black.

Walking and running are familiar and discrete gaits in humans, with distinct and contrasting underlying mechanics. Walking is characterized by, and can be defined by, relatively stiff-limbed stance phases (figure 1*a*), during which motions are largely passive, with the centre of mass (CoM) ‘vaulting’ up and over midstance under the influence of gravity (Cavagna *et al*. 1977). Running typically involves ballistic aerial phases (although many birds show ‘grounded running’, Gatesy & Biewener 1991; Abourachid 2001; Usherwood *et al*. 2008) with spring-like (but possibly ‘pseudo-elastic’ (Ruina *et al*. 2005)) stance periods (figure 1*c*).

Walking at high speeds (Alexander 1980, 1995)—and particularly at high step lengths (Usherwood 2005)—is prohibited by the failure of gravity to provide the centripetal acceleration required for an arcing vaulting path of the CoM about the stance foot; walking legs cannot be loaded in tension. However, this does not account for why running is not favoured at low speeds. Recently, numerical energetic optimizations of reductionist models (initially with point-mass and purely telescoping leg; Srinivasan & Ruina (2006, 2007) but now with developing complexity, Srinivasan (in press)), have considered a huge range of potential bipedal gaits, and demonstrated that walking and running should be favoured at certain speeds and step lengths. Without biologically realistic constraints on limb forces, ‘*impulsive*’ walking and running (involving near-infinite limb forces) are found to be least costly—in terms of energy—at low- and high-speeds, respectively. Interestingly, Srinivasan and Ruina's optimization predicts, at intermediate speeds and particularly at long step lengths, a gait currently unrecognized in the biological literature (figure 1*d*). This is described as a ‘hybrid gait’ or a ‘pendular run’.

To highlight the distinction between un-inverted and inverted-pendular mechanics, I qualify the description, thus referring to the hybrid gait as an ‘inverted pendular run’. This gait involves both a walking-like vaulting stance phase with impulsive telescoping powering at the end of the stance, and a ballistic aerial phase (figure 1*d*). However, this gait is not observed in fit adult humans, perhaps owing to the narrow predicted range of speeds at the short step lengths (relatively high step frequencies) adopted by humans (Mochon & McMahon 1980; Usherwood *et al*. 2008), or because realistic limb properties (non-infinite forces or powers) may reduce or remove the benefits of the gait.

Many non-human bipeds walk with step frequencies much closer to those consistent with a passive pendular swing-leg (Usherwood *et al*. 2008), potentially to improve stability over uneven terrain (Daley & Usherwood in press). At these relatively large step lengths, there is a much greater range of speeds for which inverted pendular running is predicted. The present study reports CoM motions of guineafowl and pheasants derived from forceplate measurements to demonstrate the presence of a hybrid gait at close to the speed/step length initially predicted by the computer optimization of Srinivasan & Ruina (2006), and here supported by simple mechanical considerations.

## 2. Material and methods

### (a) Hodographs

**Figure 2.**
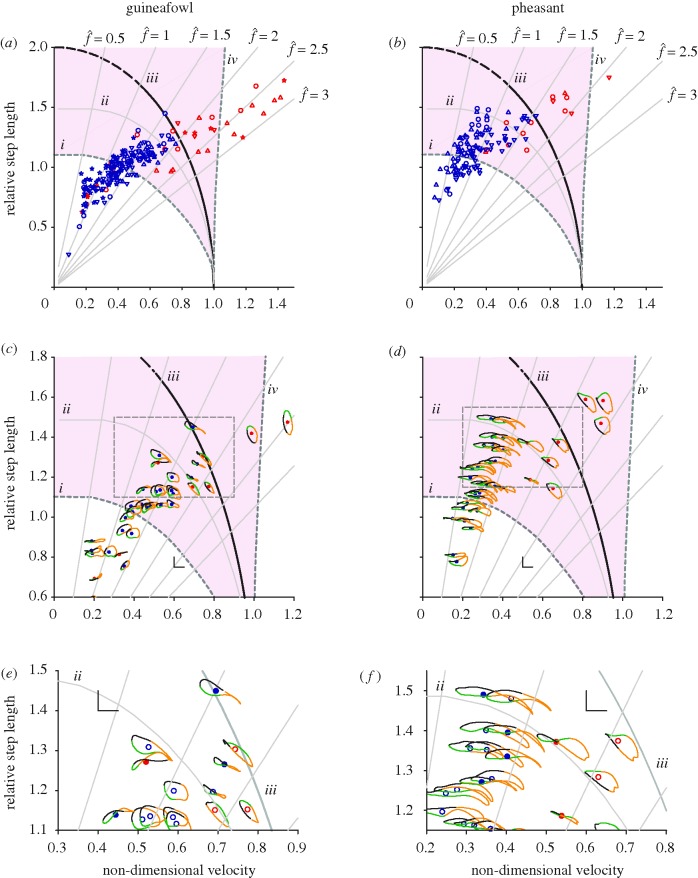
Forceplate-derived results for steady locomotion in guineafowl ((*a*) four individuals coded by symbol shape; (*c*,*e*) one individual) and pheasants ((*b*) three individuals coded by symbol shape; (*d*,*f*) one individual). Net hodograph sense (*a*,*b*) provides an objective—if arbitrary—metric for gait transition between predominantly walking (anticlockwise, blue) and running (clockwise, red). 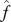 values indicate multiples of step frequency consistent with pendular swing-leg timing and duty factor = 0.5. The region shaded pink (between contours *i* and *iv*) is that predicted by numerical optimization to favour (energetically) inverted pendular running (Srinivasan 2006). The grey contour (*ii*) indicates the maximum boundary for a passive, stiff-limbed vaulter in which gravity provides sufficient centripetal acceleration of the CoM towards the foot to keep the leg under compression. The outer, black curve (*iii*) indicates the walk–run (run–walk) transition boundary predicted from energy minimization derived from collision mechanics *while allowing limb tension*. Hodographs from a single guineafowl (*c*,*e*) and pheasant (*d*,*f*) show the progression from midstances (black/green boundary) through single steps (order: green, orange, black). The right angle (*c–f*) gives a 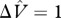 scale for the hodographs. Grounded inverted pendular running occurs near the grey walk-limit boundary, as indicated by the highlighted figure-of-eight hodographs ((*e*,*f*) providing a magnified view of the dashed box regions in (*c*,*d*)).

The walk–run transition in many birds is much less discrete than in adult humans, with running including completely unsupported aerial phases often occurring only at very high speeds. The transition between walking and running mechanics has been considered as blurred, with ‘grounded running’ describing running mechanics without any aerial phase, and little in the way of discrete transitions in kinematics (Gatesy & Biewener 1991; Gatesy 1999) or energy recovery (a metric commonly used to indicate passive vaulting mechanics, Cavagna *et al*. 1977; Heglund *et al*. 1982; Usherwood *et al*. 2008). An alternative representation of whole-body gait mechanics, not wholly divorced from the concepts of energy recovery, can be achieved with ‘hodographs’ (Kuo 2001; Ruina *et al*. 2005). Hodographs show velocity trajectories through time, and can represent the instantaneous values of horizontal (*V*_*x*_) and vertical (*V*_*y*_) velocity of the CoM at every point throughout the gait cycle (figures 1*b,c*, *e,f,h,i* and 2*c*–*f*). Idealized walking mechanics results in an anticlockwise hodograph; running with a clockwise sense; and the hypothetical ‘inverted pendular running’ gait is a horizontal figure-of-eight, with the anticlockwise component to the left (slower), and the clockwise to the right (faster).

### (b) Models

The limiting boundary for stiff, passive compass-gait walking (grey curve *ii*, figure 2) is as derived in Usherwood (2005) and more thoroughly investigated in Srinivasan (2006). This model takes account of both the increases in CoM speed, and the reduced component of gravity in line with the leg, at the extremes of stance and assumes that leg tension cannot be achieved. Hence, faster walking requires shorter steps.

The black boundary (*iii*, figure 2) marks walk–run (run–walk) transition conditions predicted from energy minimization based on collision mechanics of impulsive walking (following the principles of Kuo (2002), specifically the separated toe-off-heel-strike scenario of Ruina *et al*. (2005, fig. 5)) and impulsive running (Rashevsky 1948; Ruina *et al*. 2005; Daley & Usherwood in press). Fluctuations in CoM speed throughout the vault are not accounted for; the boundary is least precise with long steps. In this model, leg tension *is* permitted during walking. In effect, impulsive running is favourable at high speeds and long step lengths because the collisions from one ballistic phase to the next occur at relatively shallow angles; at lower speeds, impulsive, inverted pendular walking is favoured (even between the two boundaries, despite the fact that the leg is pulling the mass downward at the extremes of stance) because a large component of weight support can be achieved for ‘free’ during stiff-limbed vaulting. The analysis is not quite a simple comparison of collision angles; impulsive walking splits the collision into two smaller—and therefore less costly—collisions (figure 1*a*,*b*).

### (c) Measurements

CoM velocities of four guineafowl and three pheasants during locomotion at a range of speeds were calculated by integrating accelerations derived from forceplate (500 Hz, Kistler 9287B) measurements (Manter 1938; Cavagna 1975) following procedures approved by the Royal Veterinary College. Selected trials were steady both horizontally (maximum mean horizontal acceleration ±0.04 ms^−2^) and vertically (maximum deviation from net weight support 4%). Net changes in velocities over each step cycle were removed by mean subtraction of accelerations. Mean horizontal velocity was derived from movement of centre of pressure from midstance of one step to the next, as defined by the crossing of the horizontal force from decelerating to accelerating. Average vertical velocity was assumed to be zero.

### (d) Normalization conventions

Speeds and step lengths were normalized, allowing animals of different size to be compared conveniently with predictions from inverted pendulum and collision mechanics. Relative step length 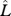 relates to step length *L*_step_ and initial leg length *L*_o_ (the height to the hip from the floor during quiet standing):
2.1
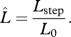

Non-dimensional velocities take account of the magnitude of gravitational acceleration *g* (taken as 9.81 ms^−2^):
2.2
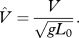

Relative step frequencies 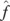 are expressed as a multiple of the frequency consistent with a passive pendular swing-leg protracting over half the stride (i.e. one step) cycle:
2.3
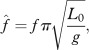

where *f* is the step frequency.

## 3. Results and discussion

The sense of the hodographs provides one objective criterion for distinguishing between ‘walking’ (anticlockwise) and ‘running’ (clockwise) gaits (figure 2*a,b*). Using this criterion, the walk–run transition does occur remarkably close to the speeds and step lengths predicted from the compass-gait model, although some running steps are also observed at relatively low speeds. However, the presence of figure-of-eight hodographs means that classifying by net hodograph sense is an arbitrary—if objective—criterion, very much akin to using a cut-off value of energy recovery (as in Usherwood *et al*. 2008).

From simple collision mechanics, walking should be energetically less costly than running (black curve *iii*, figure 2), even at speeds and step lengths that would result in leg tension (grey curve *ii*) leading to lift-off (first half of stance) or toe-dragging (second half)—effectively inverted pendular running. Computer optimization (Srinivasan 2006) predicts inverted pendular running over, and exceeding, this range of speeds, particularly at large step lengths (shaded region, from boundary *i* to *iv*, figure 2) or low step frequencies.

Figure-of-eight hodographs demonstrate that a hybrid gait does indeed occur close to the speeds/step lengths predicted by both theoretical approaches (figure 2*c–f*). These steps do not include ballistic phases—ground reaction forces never fall to zero—and so would not match the hybrid gait classification of Srinivasan & Ruina (2006). However, the figure-of-eight hodographs are broadly consistent with that of the idealized inverted pendular running gait: at midstance (black–green boundary; figure 1) the CoM is slow and in mid-vault, starting to fall; then it accelerates up and forward (green); then it falls again (orange), this time while at a higher horizontal speed, before being accelerated up while slowing, and returning to the low horizontal-speed vault (black).

The figure-of-eight hodographs therefore demonstrate a new hybrid gait—‘Grounded Inverted Pendular Running’—at a speed and step length, and with CoM mechanics, broadly as predicted from energy minimization of remarkably reductionist idealizations of bipedal gaits. The successful *a priori* prediction of this new gait supports the notion that many general aspects of gait mechanics can be understood without requiring either detailed musculoskeletal models or a presumption of obligate spring-like leg properties. It also raises the possibility that hybrid or unconventional gaits might be valuable in improving the efficiency of bipedal robots.
